# A Media Analysis of Debates on Extended Alcohol Trading Hours Changes in Scotland

**DOI:** 10.1111/dar.70251

**Published:** 2026-08-28

**Authors:** Megan Cook, Rachel O'Donnell, Gemma Mitchell, Karen J. Maxwell, Carol Emslie, Lauren Carters‐White, Andrea Mohan, Isabelle Uny, Niamh Fitzgerald

**Affiliations:** ^1^ Institute for Social Marketing and Health, University of Stirling Stirling UK; ^2^ Centre for Alcohol Policy Research, Latrobe University Melbourne Australia; ^3^ Research Centre for Health, School of Health and Life Sciences Glasgow Caledonian University Glasgow UK; ^4^ Faculty of Health Sciences and Sport, University of Stirling Stirling UK; ^5^ School of Health Sciences, University of Dundee Dundee UK

**Keywords:** alcohol trading hours, media analysis, public health, Scotland

## Abstract

**Introduction:**

Between 2017 and 2019, different policy processes in two large Scottish cities led to local authorities granting licences for later on‐premises alcohol trading hours. As a part of a wider mixed methods study, we examine news media reporting of these licencing changes to understand the content and progression of arguments at the time they were being discussed through to, and after, implementation.

**Methods:**

Forty‐four news articles were identified through online searches (published 01/01/2015‐01/01/2023) supplemented with hand searches of local publications. A qualitative thematic analysis was then conducted using NVivo.

**Results:**

Trade and local government actors were foregrounded in the media. Sectors within the alcohol retail trade responded differently, depending on their business interests. In Aberdeen, stakeholders stated that the changes were primarily intended to support businesses and in response to consumer trends. In Glasgow, stakeholders stated that the changes were needed to boost the economy and compete with other European cities. A diverse range of stakeholders framed their arguments in health terms, expressing concerns that increased hours would lead to increased consumption and harm.

**Discussion and Conclusions:**

In media reports of extended late‐night trading hours, stakeholders who were both for and against the changes put forward arguments about business and health impacts. There appeared to be little consensus about the value of extended trading hours amongst trade stakeholders who responded in line with their specific business interests, sometimes drawing on health arguments to do so. This counters the assumption of unity in trade views on liberalisation of opening hours.

## Key Point Summary

- In media reports of extended late‐night trading hours, stakeholders who were both for and against the changes put forward arguments about business and health impacts, often without underpinning evidence.
- We identified little consensus about the value of extended trading hours amongst trade stakeholders, who responded in line with their specific business interests, sometimes drawing on health arguments to do so.
- A diverse range of stakeholders framed their arguments in health terms, expressing concerns that increased hours would lead to increased consumption and harm.
- Examining the arguments made at the time the changes were being discussed, including after implementation, allowed us to capture the ongoing push for, and continual increase of, alcohol trading hours across the night‐time economy.
- Given that licencing stakeholders adopted a range of diverse arguments, there is an opportunity for public health actors to tell consistent and compelling stories to the media, and to clearly and logically refute unfounded trade claims.

## 1 Introduction

In many jurisdictions worldwide, only retail outlets which secure a licence are permitted to legally sell alcohol, under systems which have developed over time to manage the harms associated with alcohol availability. In Great Britain (England, Wales and Scotland) national legislation sets out a policy framework that is implemented by local authorities [1, 2]. Between 2017 and 2019, two different licencing processes led to local authority decisions to permit later alcohol trading hours for on‐premises outlets in two large Scottish cities—Glasgow and Aberdeen. In 2019, 10 nightclubs in Glasgow were granted a one‐hour extension to their alcohol trading hours, allowing them to open till 4 am, as a part of a 12‐month pilot scheme planned by the Glasgow Licensing Board [3]. As part of the pilot, the board granted the extra hour to premises deemed to meet specific criteria, including security and safety measures for both customers and staff, designed to promote the national statutory licencing objectives. In Scotland, these objectives are *preventing crime and disorder*, *securing public safety*, *preventing public nuisance*, *protecting and improving public health* and *protecting children and young people from harm* [2, 4]. In Aberdeen City, 38 licenced venues were granted up to two hours extension following the removal of the significant entertainment clause in the 2018 statement of licencing policy [5]. This clause originally permitted later alcohol trading hours only in premises which provided ‘significant entertainment’ (e.g., discos, DJs, snooker, adult entertainment, dancing and live music for dancing, cabaret). These changes to alcohol trading hours were implemented despite international evidence suggesting longer hours are generally associated with increased alcohol consumption and harms [6, 7, 8], and received extensive media coverage.

The media's influence on policy has been widely documented and there is strong evidence to suggest the media play an integral role in shaping debate, opinion and responses [9, 10, 11, 12]. In other words, the media can be considered as active participants in the social construction of problems and responses to them. Research on agenda‐setting argues that news media exert influence on public attention by selecting which stories to cover and how much salience they are afforded [13]. Beyond agenda‐setting, news coverage also frames social issues in particular ways, selectively emphasising certain interpretations, causes and solutions while marginalising others [14]. Importantly, the perspectives represented in news media are themselves shaped by sourcing practices and the strategic interventions of stakeholder groups, including governments, industry actors, advocacy organisations and researchers [12, 15]. Indeed, previous media analyses suggest that the alcohol industry has become more effective at using media channels to communicate (including promoting certain arguments) to a wide audience and lobby for action [16, 17]. For example, a recent study found Finnish news media outlets prioritised politicians and alcohol industry voices when reporting on the 2018 *Alcohol Act* reform [18]. Another Finnish study suggested the media frequently adopt positions which align with the alcohol industry's interests, with almost half the editorials identified in their sample arguing against alcohol availability regulations, and pushing for liberalisation [19]. Analysing news reporting therefore provides insight not only into the content of policy debates in the media, but also into the ways in which particular perspectives become amplified and how policy responses come to be enacted [12, 15, 20].

In comparison to a growing body of media research focussed generally on alcohol [21, 22, 23, 24, 25], explorations of news media coverage on alcohol licencing are more limited and have predominantly focussed on the Australian and UK contexts [15, 20, 26, 27]. For example, Cook and Wilkinson [27] argue that media reporting contributed to a policy environment where public perceptions of a threat to live music venues became a barrier to restricting hours of sale in the future. Additionally, in the UK, a recent analysis identified how news media framings of alcohol licencing policy during COVID‐19 shifted from highlighting ‘risky’ premises to ‘problematic’ policy‐making [28]. Thus, the different licencing changes in Glasgow and Aberdeen present a unique opportunity to consider media coverage in Scotland, a context rarely considered in previous media coverage of alcohol licencing, and specifically alcohol trading hours. As a part of a larger mixed methods study, Evaluating Later and Expanded Premises Hours for Alcohol in the Night‐time Economy (ELEPHANT), which aimed to understand and evaluate the introduction, operation and impact of later trading hours for licenced alcohol premises in Glasgow and Aberdeen (see also [29, 30, 31]), the aim of the current study is to examine reporting of the extensions in alcohol trading hours in news media to understand the content and evolution of arguments for or against the changes at the time they were being discussed through to, and after, implementation. Because other elements of the larger ELEPHANT study are retrospective, this analysis also aims to contribute to an understanding of how the changes came about, by examining who was driving the discourse in the media, and how, at the time the changes were being considered and implemented. In doing so, we aim to enhance understanding of how policy debates unfold, who participates and the processes through which specific policy responses emerge [12].

## 2 Methods

### 2.1 Study Design

We conducted a qualitative thematic analysis of news media articles [32]. To study the content and evolution of arguments for or against the extensions in alcohol trading hours in Glasgow and Aberdeen, our approach draws on the work of Hansen and Gunter, which is useful for mapping a social issue's manifestation and development by identifying key stakeholders (including industry, public health advocates and the general public) to understand who and/or what drives the discourse [12].

### 2.2 Search Strategy and Screening of Articles

Two separate online searches of Lexis Nexis were conducted in mid‐2023 to obtain articles reporting on the alcohol licencing changes in Aberdeen and Glasgow. Several search strings were piloted using a range and combination of city, licencing, temporal availability and alcohol terms. The final search terms, developed by the first author [MC] in consultation with an information specialist and NF, were:

1. (Aberdeen OR Grampian) AND (‘significant entertainment’ OR 3 am OR ‘extended trad*’ OR terminal OR ‘terminal hour’ OR ‘closing time’) AND (alcohol* OR drink*) AND licen*
2. Glasgow AND (‘pilot scheme’ OR 4 am OR ‘extended trad*’ OR ‘terminal hour’ OR terminal OR ‘closing time’) AND (alcohol* OR drink*) AND licen*

**FIGURE 1:**
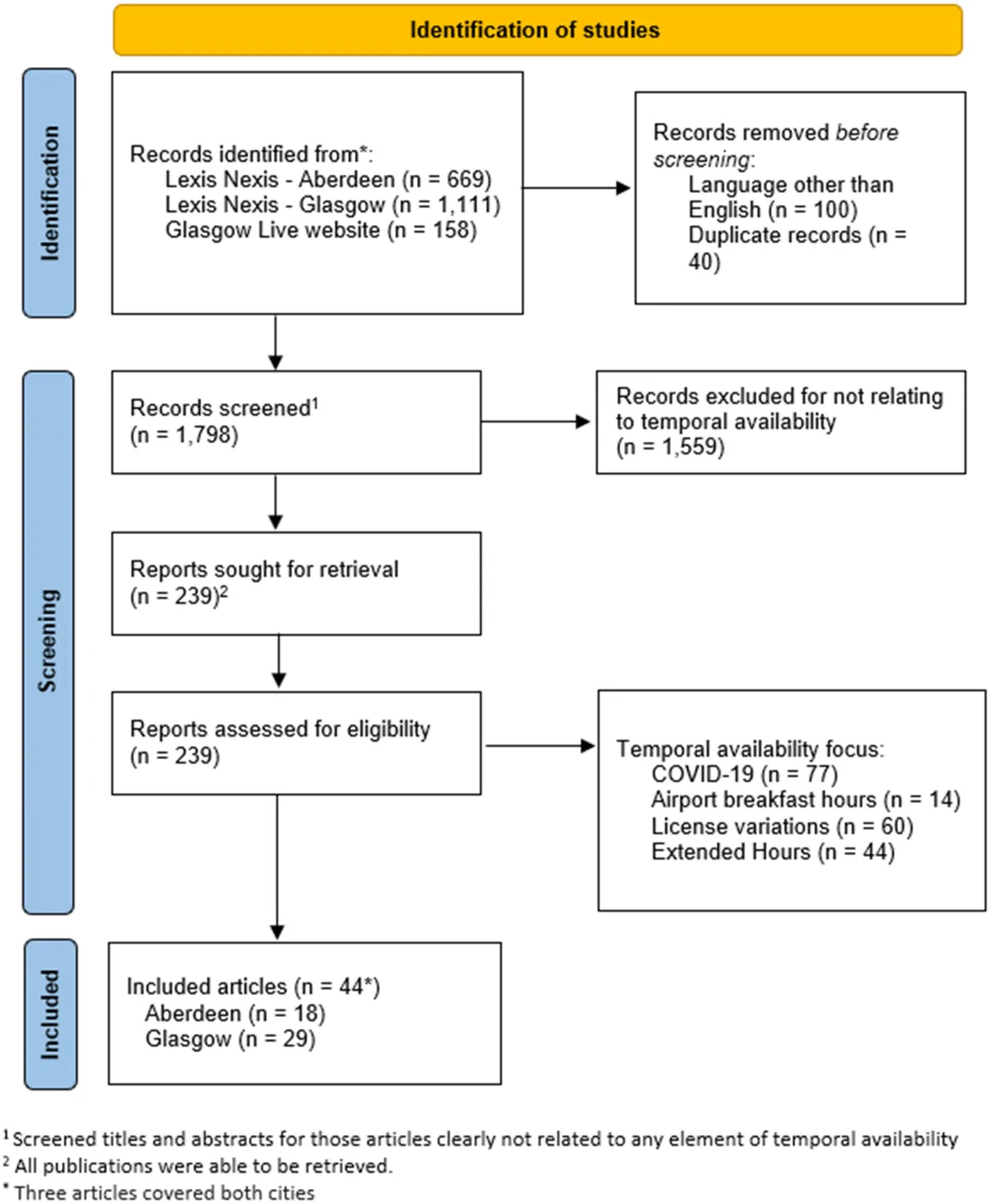
PRISMA search tree.

We restricted the searches to 01/01/2015‐01/01/2023 to capture early discussions of the changes as well as the evolution of those discussions over time as the policy changes progressed. The first searches identified 1780 relevant articles. To capture smaller local newspaper reporting not available via Lexis Nexis, the above searches were supplemented with a hand search of local papers' web archives (using the same search terms and time period). This resulted in 158 new articles, giving a total sample of 1938 papers. After removing duplicate records (40) and those in a language other than English (100), we were left with 1798 articles to screen. Screening was conducted by MC based on titles and full text of articles. Articles were included if they discussed the local late‐night trading hour changes in Aberdeen or Glasgow, but excluded if another aspect of temporal availability was discussed (e.g., changes to temporal availability in response to COVID‐19 mitigation measures). The final data set comprised of 44 articles (see Figure 1).

### 2.3 Analysis

Articles were imported into QSR NVivo 20 for coding and analysis. A coding structure was deductively developed. Deductive coding involves applying a set of pre‐determined codes to the data, and in our case, this was based off previous literature. Firstly, we drew on previous media analyses [23, 24] and added codes to capture basic descriptive information including newspaper and type of article. Next, we drew on the framework proposed by Hansen a Gunter [12] as a guide. Hansen and Gunter describe the importance of capturing who the key claims‐makers are, and our coding framework made sure to code sources in detail (e.g., coding different types of trade stakeholders—see below). We also remained attentive through the analysis process to who is making the claims.

The deductive coding framework provided a systematic foundation grounded in the previous literature and allowed us to identify and categorise key features of the news articles. For each article the first author coded the *type of article*, *publication date, newspaper*, *stakeholders quoted*, *temporal availability focus, proposed outcomes, arguments for the extended alcohol trading hours, arguments against the extended alcohol trading hours changes* and *other*. We divided the quoted stakeholders into eight groups^1^: (i) business advocacy organisations (organisations which advocated for all business interests, not just licenced trade); (ii) trade and industry bodies (this included individual operators, venue managers, as well as trade bodies representing operators); (iii) police; (iv) local government—including elected members (known as ‘councillors’) and staff; (v) general public; (vi) public health—including doctors and alcohol charities; (vii) solicitors; and (viii) other.

After the initial coding was complete, the first author reviewed all the coded data and drew these together into higher‐order conceptual categories which aimed to identify the key arguments. For example, from the coded data on supporting the changes, the higher order categories captured the reasons why the changes were supported, for example, extended hours were what consumers desired, would lead to profit for business and would support cultural competitiveness. Practically this resulted in several documents where we grouped all arguments for the changes relating to consumers, all arguments for the changes relating to economic benefits etc. and did the same for those arguments against the changes and for each city. These conceptual categories (or groups) form the key arguments we overview in the findings.

Given this study is part of a larger project, and to inform future policy and research, it was also important that we not only summarise the media discourse but also provide deeper analytical insight and draw out meaning from the data. Thus, a further stage of thematic analysis then examined the coded data for patterned responses, as well as points of tension. This involved the first author going back and re‐reading all the coded and categorised data, along with notes taken during the process to identify patterns and tensions across these arguments. The first author discussed the findings and the initial themes with the team, and we settled on two final themes. We begin by overviewing the descriptive characteristics of the articles and the key arguments made in each locality.

## 3 Results

The changes in local alcohol trading hours policy in Aberdeen were a focus in 18 articles, while the changes in Glasgow were a focus of 29 articles (three articles covered both). Of those 44 articles, 33 were standard news articles, 11 opinion pieces or letters, two editorials and one feature article, and the majority were published in 2018 (*n* = 30). As can be seen in Table 1, which provides an overview of the article's descriptive characteristics, there were several common stakeholder groups quoted in the news media in both cities, however key differences in participating stakeholders were also identified. For example, in Aberdeen local advocacy organisations (e.g., a heritage association and Aberdeen Inspired) were a unique group participating in the media (i.e., they were not identified in articles on the changes in Glasgow). In Glasgow, representatives for a transport group, Glasgow City Health and Social Care Partnership, national public health advocacy organisation Alcohol Change UK, street pastors and representatives of staff in the late‐night economy were also quoted (these stakeholders were coded in the ‘Other’ category).

**TABLE 1:** Article characteristics.

	Aberdeen	Glasgow	Total
No. of articles	18	29	44^a^
Type			
News	11	22	33
Opinion	7	4	11
Editorial or feature	0	3	3
Year			
2018	10	20	30
2019	5	3	8
2020	3	3	3
2022	0	3	3
Stakeholders^b^			
Business advocacy organisations	3	0	3
Trade and industry bodies	6	14	20
Other	4	6	10
Police	1	8	9
Local government	8	19	27
General public	7	3	10
Public health	1	5	6
Solicitors	1	2	3
Publication			
Sunday Mail	0	1	1
Glasgow Live	0	4	4
Evening Times	0	9	9
Aberdeen Evening Express	10	0	10
The Herald	0	5	5
Aberdeen Press and Journal	5	0	5
Scotsman	0	2	2
Daily Record	0	1	1
Metro (UK)	0	1	1
Impact News Service	2^a^	2^a^	2^a^
ENP Newswire	1^a^	1^a^	1^a^
Scottish Daily Mail	0	2	2
The Times (London)	0	1	1

^**a**^ Three articles covered both cities.

^b^ This is the number of articles which included a quote from each stakeholder group.

Most news articles outlined the nature of the changes being proposed, and included quotations from stakeholders amongst whom there appeared to be little consensus about the value of extended trading hours. Few quoted stakeholders were neutral about the changes (only police presented neutral statements to the media). In the following sections we begin by overviewing the key arguments made in each locality (to identify differences in the arguments being used in Aberdeen and Glasgow). This is followed by examining which stakeholders were making these arguments and examining points of alignment (where different stakeholder groups are drawing on the same arguments) and contention (where stakeholders from the same groups are making contesting arguments).

### 3.1 Overview of Arguments

In Aberdeen, those in *support* of giving bars similar alcohol trading hours to nightclubs made three main arguments; firstly, that the change was what consumers desired or ‘reflect what folk in Aberdeen want’ (*Aberdeen Evening Express*, 9 November 2018). Secondly, that the extension in alcohol trading hours would increase consumer choice and diversity in the late‐night economy:

I think it's fair to say that people are looking for a different kind of experience now, particularly around the hybrid type venues, which provide food and drink and perhaps some live music compared to the traditional nightclub environment. Although, I'm sure there will still be those who prefer the nightclubs, our city should be able to cater for all. (*Aberdeen Evening Express*, 9 November 2018).

Thirdly, those in support argued that the changes would have business related benefits, supporting a struggling sector by allowing ‘entrepreneurialism and commerce to develop’ (*Aberdeen Evening Express*, 3 November 2018). Collectively, these arguments reflect a market‐oriented framing of the night‐time economy, in which the licencing changes were justified through the language of consumer choice, entrepreneurialism and competition.

Those *against* the extension to alcohol trading hours in Aberdeen made three main arguments. Firstly, there were a series of economic and cultural concerns about the decline of traditional nightclubs and the erosion of Aberdeen's nightlife ‘vibrancy’, positioning the licencing changes within a broader debate about urban identity and the future of the night‐time economy. For example, arguing that ‘the proposal could “kill off the traditional nightclub”’ (*Aberdeen Evening Express*, 12 September 2018). Relatedly, some also argued that the lack of diversity and vibrancy would mean that people would go elsewhere for a night out: ‘It will get to the point where Aberdeen could just end up as a city of bars with people going to other cities such as Edinburgh for a more vibrant experience’ (*Aberdeen Evening Express*, 10 January 2019). Additionally in one article concerns were raised that this policy change would create a price war with different venues trying to undercut each other to attract clientele. The second key argument was that the changes would lead to greater alcohol consumption and alcohol‐related health harms, as well as a rise in antisocial behaviour and risks to public safety:

If the number of late‐night‐opening premises increase, without there being a requirement to provide suitable entertainment, it is likely there will be an increase in late‐night consumption of alcohol and more public crime and disorder, less public safety and an exponential increase in public nuisance when patrons of these premises and legitimate late‐night premises empty on to the streets. (*Aberdeen Evening Express*, 3 November 2018)

Finally, those against argued that increased trading hours would increase burden on services such as the National Health Service [NHS], public transport, door stewards and police:

The NHS, police and other agencies are already overstretched. This will only make matters worse. (*Aberdeen Evening Express*, 8 December 2018).

Through the amplification of these arguments, news reporting in Aberdeen contributed to an understanding of the extended licencing trading hours as focussed on risk, disorder, urban decline and market forces.

In Glasgow, those in *support* of an extension in alcohol trading hours to 4 am for nightclubs made five key arguments, particularly focussing on extended trading hours as a mechanism for urban economic development, modernisation and cultural competitiveness. Firstly, those in support argued that the licencing changes would result in improved access to transport (i.e., ability to catch the first train home at 5 am). Secondly, that the changes would have economic benefits supporting the city centre economy and businesses: ‘*Glasgow licensing chiefs said that allowing late‐night venues to remain open an extra hour could create jobs.’* (*Scottish Daily Mail*, 16 August 2018). An unpublished report commissioned by Glasgow City Council was regularly cited, which claimed that Glasgow's night time economy generated around £2.16 billion annually and supported 16,000 jobs. Thirdly, that the changes would allow Glasgow to compete with other European cities, making the city feel more cosmopolitan and attractive:

Club owners say the city's current licensing policy is ‘restrictive’ and it is hoped adopting a more European approach to opening hours will allow Glasgow to compete with continental destinations such as Barcelona and Berlin. (*Evening Times*, 16 August 2018)

In doing so, this argument constructed extended opening hours as markers of urban vibrancy and, unlike Aberdeen, associated this with international standing. Fourthly, arguments that these changes would mirror the perceived ‘relaxed drinking laws of Europe’ were also drawn on to suggest potential benefits by tackling the rush to consume alcohol at the end of the night: ‘Club owners backed the plans and said adopting the flexible approach to opening could help tackle the rush to consume alcohol.’ (*Evening Times*, 16 August 2019). This directly challenges arguments that increased availability produces greater risk (see below). Finally, it was argued that the changes would reward those who invest in safety, further incentivising good practice:

It is only nightclubs which can apply for the pilot. It will reward those who invest in safety. (*Evening Times*, 8 November 2018)

Arguments that the policy would ‘reward’ venues investing in safety also shifted responsibility for managing alcohol‐related harms onto individual businesses, positioning well‐regulated operators as responsible partners in urban governance rather than contributors to disorder. Taken together, these patterns suggest that supporters strategically combined economic, cultural, and public safety narratives to construct extended trading hours as both commercially beneficial and socially responsible.

In Glasgow, those *against* the changes made four key arguments. Firstly, that the changes would result in increased alcohol consumption and health harms:

I thought the recent introduction of minimum pricing which led to an increase in the price of alcohol was to stop people drinking too much. How can opening until 4am be good for anyone except for bar takings and tax revenue? (*Metro* [UK], 17 August 2018)

In doing so, those against the changes positioned the policy as contradictory to public health. Secondly, it was argued that the changes would lead to increased noise and disruption: ‘The idea of 4am licences will also be a particular concern to residents who live near clusters of pubs and clubs–quite naturally, they will worry that longer opening hours can only mean one thing: more noise and disruption’ (*The Herald*, 16 August 2018). These concerns regarding noise, disruption, and pressures on nearby residents also reflected longstanding tensions surrounding the night life governance [33]. Thirdly, that the changes would result in increased costs and staffing issues for licenced premises, exacerbating an already existing issue: ‘*Our [trade] members tell us there are transport problems and there will be difficulty getting staff to work the extra hour’* (*Evening Times*, 8 November 2018). In linking to institutional vulnerability these arguments expanded the debate beyond individual drinking behaviour to the wider infrastructural demands of the night‐time economy. Finally, it was suggested that the changes would increase the burden on services including health, transport, police and others responsible for the welfare of those in the night time economy (e.g., street pastors):

Most folk in the trade that I've spoken to don't want it. Where are the resources for the police and the NHS? There is a big problem here (*Evening Times*, 13 September 2018).

Through the amplification of these arguments, media reporting in Glasgow contributed to framing extended licencing changes through a lens of risk (whether that be to lifestyle, health, economy etc.).

Later articles included discussion of a further change in trading hours policy in Glasgow, under a new pilot which would allow Glasgow pubs to apply to open until 1 am (instead of midnight). Those in favour of the proposal suggested it would support the late‐night economy and provide a better experience for customers: ‘*That's why we believe an extra hour for city centre bars, maintaining our vibrant club scene and attracting more families into the city centre after retail hours makes a stronger offer for the customers and for businesses.’* (*Evening Times*, 29 December 2022). When supporting the changes, stakeholders suggested that staggered hours could be beneficial because they do not place the same ‘pressures’ on public transport: ‘At present, there is a compelling case that more staggered exit times from city centre premises may not place the same pressures on transport availability.’ (*Evening Times*, 29 December 2022). But sources within the hospitality trade said extra hours might not necessarily bring benefits due to increasing costs and staffing issues already faced by publicans.

We now examine which stakeholders were making these arguments and critically contrast the arguments being put forward in both Aberdeen and Glasgow to explore the content and evolution of stakeholders' sentiment and to identify alignment, inconsistencies and tension across the media commentary.

### 3.2 Alignment of Stakeholder Arguments

Arguments used to support positions for or against the changes aligned in unexpected ways between different stakeholder groups. Firstly, arguments were framed in health terms by multiple stakeholders including those from trade backgrounds. In Aberdeen, several trade stakeholders (nightclub operators and venue owners) argued that changes undermined public safety and made arguments focused on public health, including a likely increase in public drunkenness and violence, to oppose the changes. For example, nightclub owners claimed extended hours would lead to increased consumption:

In nightclubs drinking is broken up with dancing and listening to music. This [policy change] would encourage more alcohol consumption. (*Aberdeen Evening Express*, 10 January 2019).

The mobilisation of public health and safety arguments by trade stakeholders is particularly notable because it complicates conventional assumptions that commercial actors uniformly oppose health‐oriented framings of alcohol policy. Members of the general public similarly drew attention to the significant alcohol‐related health harms Aberdeen already experiences to argue against the changes, and in one article the NHS were noted as having concerns about the changes due to the ‘*health implications*’ (*Aberdeen Press and Journal*, 9 November 2018).

In Glasgow, discussion of a European approach to licencing was a point of alignment and tension. Firstly, several diverse stakeholders drew on arguments about a European approach to licencing to justify the changes. For example, the pilot was described by one reporter as ‘mirroring the more relaxed drinking laws of Europe’ (*Evening Times*, 16 August 2019). The appeal of this European approach was the perceived positive impact on alcohol consumption and public nuisance, and about ensuring Glasgow aligned itself with comparable European cities, with nightclub owners and local government seemingly aligned on this argument. For example, government stakeholders were reported as saying that ‘there's a strong view that a more European approach would avoid the rush to consume alcohol before closing time that then spills out on to our city centre streets.’ (*The Herald*, 16 August 2018). In this way, the ‘European’ framing enabled stakeholders to combine economic, cultural, and public health rationales in support of deregulation. However, tension was identified, with several stakeholders aligning to argue against this thinking, proposing that there is nothing European about the changes:

There is nothing remotely European about any of this. Keeping the clubs open for another hour will only make it more British. (*Scottish Daily Mail*, 17 August 2018)

A medical doctor and director of an alcohol non‐profit organisation also attempted to counter the European narrative by suggesting this approach was not viable in Scotland and that increased restrictions and regulation were necessary for addressing increasing consumption and harms. One member of the public also countered the view that longer hours would lead to less alcohol consumption and harms in a letter to the editor, saying;

I NOTE the fairy tale about 4am drinks licences leading to less drunkenness […]. The excuses are the same every time licences are extended taken from Ye Old Handbook of More Profits. Let's be blunt – if you can stand up you will be served alcohol. Rather uncomfortably, drunkenness and the like has risen with all these lovely progressive sophisticated opening hours. (*The Herald*, 20 August 2018).

The identification of this line of argument suggests that stakeholders were not simply debating licencing hours themselves, but competing over broader narratives concerning the desired character of urban nightlife.

Compared to Aberdeen where public health stakeholders were less visible in the news media, in Glasgow a representative from Glasgow City Health and Social Care Partnership drew on evidence around greater availability leading to increased health harms to oppose the changes, suggesting that ‘restricting hours is one of the ways of reducing the harm associated with excess consumption’ (*Glasgow* *L* *ive*, 16 May 2019). Other public health actors, including doctors, were also reported as drawing on research evidence around pre‐existing levels of harm which they argued were already too high, with data provided on the rates of alcohol‐related problems experienced in Glasgow and the number of people using late‐night services, such as street pastors. Again, there was alignment with members of the public quoted in the articles in Glasgow, who also raised concerns about health harms.

### 3.3 Contentious Perspectives

The multiple perspectives and actors evident in the debate allowed us to identify contention within stakeholder groups across the media commentary. In Aberdeen, local government, advocacy and trade (venue owners) in support of the extended alcohol trading hours changes emphasised the economic benefits to the broader night time economy and city centre, for example:

This decision is going to give businesses within the city centre the option to diversify their offering. Our city centre is changing and this decision will play a part in enhancing potential for businesses, while also keeping pace with developments and growth within the city. (*Aberdeen Press and Journal*, 14 November 2018; Advocacy).

A large hospitality operator also argued that the changes would facilitate business growth and innovation. However, this economic argument was not supported by all trade commentators in Aberdeen. For example, nightclub venue owners and a trade association claimed the extended hours would lead to venue closures and job losses because of increased competition and be the ‘*death knell*’ for the nightclub industry as well as negatively affect the overall vibrancy and diversity of the city, leading to customers going elsewhere (*Aberdeen Evening Express*, 10 January 2019). Overall, such trade arguments against the changes dominated news media reporting in Aberdeen. These tensions between trade stakeholders suggest that ‘industry’ was not represented through a singular economic interest. Rather, different commercial actors strategically mobilised competing narratives depending on how the proposed changes aligned with their business models. This highlights how this media debate around extended hours policy became a site where competing commercial interests attempted to establish their preferred vision of the night‐time economy as economically legitimate and socially desirable.

We also identified contentious perspectives amongst government stakeholders in Aberdeen, with a small group arguing that nightclubs required more support from government, while others adopted a free market perspective: ‘the market would decide how successful firms would be and it wasn't for the council to set policies to protect nightclubs’ (*Aberdeen Press and Journal*, 9 November 2018). Such divergent positions within local government commentary likely reflect broader ideological tensions between stakeholders. During the public consultation in Aberdeen, local government commentators were reported as saying that consideration of later hours was a response to consumer trends (including young people being less likely to attend nightclubs) and reshaping the night‐time economy towards hybrid venues to reflect consumer demands. Yet, members of the public commentating in the media during 2018 expressed limited support for longer hours. While public commentators may not be representative of wider public views, members of the public quoted raised concerns about the existing level of alcohol‐related harms in Aberdeen, including economic costs, public nuisance (specifically street drinking), and burdens on services such as police, health, and social care (see also [3, 29]). There were concerns that many public services (including NHS and police) were already overstretched and the extended hours will intensify existing strains. Reported comments from the public suggest that they perceived the changes would lead to increased antisocial behaviour and public disturbance, which would be felt most keenly by city centre residents (see also [29]).

In Glasgow, however, reports on the public's views were more mixed. Similar to Aberdeen, some members of the public disagreed with the changes, focusing on the balance between public health and profit which they believed the licencing board had got wrong. For example,

I thought we were trying to reduce the consumption of alcohol and reduce the incidence of drink‐fuelled violence. Clearly business has more say than the police and the public. (*Daily Record* and *Sunday Mail*, 8 November 2018).

However, several members of the public were reported in the news media as being positive about the changes as it would mean more time spent out in the night time economy: ‘Woohoo, even more reason for me never to leave the city.’ (*Daily Record and Sunday Mail*, 8 November 2018).

In both Glasgow and Aberdeen, concerns were raised by several different stakeholders that the extended alcohol trading hours would have impacts on wider services which wind down after 3 am. In Aberdeen, venue owners were amongst those arguing that longer alcohol trading hours would be detrimental to police, transport or other agencies, and as a result put people's safety at risk. In Aberdeen, concerns were also raised by nightclub operators about a shortage of properly trained door stewards as a result of extended alcohol trading hours. In Glasgow, trade associations, venue owners, and street pastors referred to the burden to health services from existing levels of alcohol consumption and the industry's contribution to levels of harm – ‘This harm goes far beyond street drinking to higher levels of alcohol‐related health conditions, hospital admissions and more’ (*Scotsman*, 6 November 2018; Advocacy). However, some government stakeholders in Glasgow emphasised the nature of the pilot was to assess the wider implications on service provision. They also consistently referred to the ‘safeguards’ and standards pilot participants would need to meet. Despite this, in September 2018, street pastors expressed ‘fears’ about the extended alcohol trading hours changes, and the extra workload and burden on their services:

It's fine to extend the opening hours but it has implications for others who serve the city centre. Fast food outlets, those cleaning the streets and the street pastors. We [street pastors] will have to think about changing our opening hours. We will see the same issues but an hour later. There is the potential for those who do misuse alcohol to misuse it further. (*Evening Times*, 8 November 2018).

These comparisons between Aberdeen and Glasgow suggest that media debates surrounding the licencing changes were shaped by differing local narratives regarding the purpose and identity of the night‐time economy.

## 4 Discussion

This analysis of news media reporting of the extended alcohol trading hours changes in Glasgow and Aberdeen identifies the range of arguments being put forward publicly for or against the extended hours changes at the time they were being discussed. Overall arguments predominantly focussed on business/economics, night time economy diversity, public safety and health, and consumer demands. There were some differences in arguments between the two localities, notably that those in favour of the changes in Aberdeen, but not in Glasgow, argued that customers wanted the changes. In Glasgow a unique argument centred on making the city more ‘European’. Overall, the key arguments identified in both localities—a focus on public safety and public health, sustaining the night time economy culture in light of other offerings and arguments related to business viability—are consistent with those found in previous analyses of news media on liquor licencing suggesting some common patterns in global arguments related to alcohol licencing [20, 26, 27].

Our analysis illustrates how diverse stakeholders within the alcohol retail trade responded differently to the specific proposals in each city, depending on their business interests. In Glasgow, where the proposed liberalisation involved extra trading hours for nightclubs only, we found that nightclub owners argued in favour of the changes, while trade associations and other venue owners were seemingly against. In Aberdeen, where only bars were being granted extra hours and would now compete for customers at the same time as nightclubs, bar managers and owners supported the increased hours, while nightclub operators were against. This diversity of views within the alcohol industry is similar to that found in analysis of alcohol industry attempts to frame debate about pricing policy [11], and industry views on cumulative impact policies [32]. Some trade actors argued against liberalisation because it would lead to greater competition for their businesses. Vehement opposition to any changes which increase competition was also identified in a recent review of the Northern Ireland licencing system [34]. More broadly, this also suggests that media debates surrounding extended trading hours were not simply disputes over licencing policy, but contests over the future character and purpose of the night‐time economy itself.

Similar to a recent Finnish study [18], we found trade and government actors were foregrounded (i.e., quoted more often) in debates about extended alcohol trading hours (see Table 1). Strikingly, in Aberdeen diverse trade actors framed their arguments in health terms, expressing concerns that increased hours would lead to increased consumption and harm, a view supported by research evidence [6, 7, 8]. Whereas in Glasgow, extended trading hours were proposed to help tackle the rush at the end of the night to consume alcohol in line with a perceived European approach, a view which contradicts evidence from other parts of the ELEPHANT study [35]. As such, traditional public health framing around increased consumption and harm was used to both support and oppose increased trading hours, highlighting how such arguments can be deployed to legitimise policy positions based on differing business interests [11, 36]. While acknowledging the different positions within the policy making process, the use of arguments focussing on public health by diverse trade commentators may have helped to define extended trading as not only an economic/business issue but also a public health issue, despite the less prominent role of public health actors.

In Aberdeen, public views reported in the news media were predominantly against the changes. Forthcoming analysis of consultation responses in Aberdeen also found mixed public support, with some public respondents suggesting that later trading hours would offer greater consumer choice and attract more diverse consumers to the city, while others argued that nightclubs should not be the only option for those wishing to use the late‐night economy. Across the larger ELEPHANT study, our results suggest mixed public support, rather than a clear consensus from the public that they wanted extended hours [29, 30]. It is not possible to tell from our data the basis or evidence on which some stakeholders claimed that the changes were something consumers wanted. It is possible that the media actively sought the views of vocal actors [10], and did not capture the perspectives of those lobbying behind the scenes which may have been more one‐sided. While invisible lobbying (off the public record) may not be the only factor driving policy shifts, it is part of the policy change process [37], and was likely involved in the extended hours policy changes in Aberdeen and Glasgow analysed.

Examining the arguments made at the time the changes were being discussed, including after implementation, allowed us to capture the ongoing push for, and continual increase of, alcohol trading hours across the night‐time economy. Since the initial pilot for nightclubs was announced, a new 1 am pilot was introduced in Glasgow for pubs in 2024 and the nightclub pilot was made permanent. The initial extension of trading hours for nightclubs seems to have triggered further liberalisation of trading hours, to preserve the prior three hour gap between the closing hours of pubs and nightclubs, but without any clear sense (from this data at least) of why maintaining the gap was important. Evidence from other strands of the ELEPHANT study which directly observed venues in Aberdeen and Glasgow found that many bars and clubs were not regularly utilising the late night trading hour extensions they had been granted and were relatively empty late at night [28, 34]. This calls into question the assumption that liberalisation is inevitably profitable for trade and desired by the public.

What was not evident on the part of the media was a critique of the arguments and evidence (or lack thereof) put forward by certain stakeholders. For example, claims that an extra late‐night hour for venues would attract more families to the city centre, the benefits of staggered opening hours on harms, and the benefits of adopting a European drinking culture went without critical reflection, supporting evidence, or challenge. While previous analyses have identified how the media selectively mobilise certain framings and often strategically omit certain sources to create a narrative [9], here a broad range of arguments were presented as accepted facts. In an already highly contested environment, this omission of evidence and critical engagement may further contribute to the sense of policy ambiguity. It may also be an unintentional by‐product of limitations on the resources of news media outlets.

### 4.1 Limitations

These findings need to be considered in light of several limitations, firstly, print news coverage represents just one part of the range of information sources to which people might turn and we are unable to determine from the sample the role of invisible lobbying (in other words we were constrained by the documentation obtained and the media reporting available). As such, although the news media provides lens as yet uncaptured by other elements of the larger study, it is necessarily an incomplete picture of commentators and arguments. Secondly, with the growth in online and particularly social media, traditional print news media has been suggested to have lost some of its power in shaping the news landscape. However, according to a 2023 Ofcom survey [38], print news media continues to be consumed by around 26% of UK adults 16+, and studies of the agenda setting influence of traditional news media overtime have found little evidence declining influence [39]. Thirdly, the media are not unbiased and are shaped by political powers including ownership. While we noted in Table 1 the publications identified, we did not examine whether arguments differed based on media outlets or ownership, this may be worthy of consideration in future research. Moreover, reporting may also be shaped by stakeholder intervention, and this bias may be reflected in the arguments and number of stakeholders identified in our study (e.g., the predominance of the alcohol industry and the relative omission of public health stakeholders). However, given other elements of the larger ELEPHANT study struggled to engage alcohol industry representatives, capturing a range of industry views may also be considered a strength within the larger study. Finally, the search strategy may have excluded relevant publications. However, it was developed in consultation with the larger research team and piloted to ensure appropriate coverage.

## 5 Conclusion

In conclusion, in media reports of extended late‐night trading hours for licenced premises, stakeholders who were both for and against the changes put forward arguments about business and health impacts. We found that trade representatives are not universally in favour of greater trading hours for licenced premises: some oppose liberalisation when it will lead to greater competition (such as in Aberdeen media reports), and then employ a range of public health focussed arguments about increased harms, while others have concerns about the costs of opening later (as seen in Glasgow media reports). Additionally, in Aberdeen, support for the extended trading hours seemed to come not only from trade representatives who stood to benefit, but to rest on local government beliefs that the changes would bring economic benefits, but without robust analysis or evidence of how that would happen. Given that it seems as if licencing stakeholders lack consistent arguments, there is an opportunity for public health actors to tell consistent and compelling stories to the media, and to clearly and logically refute unfounded trade claims [40].

## Author Contributions

Each author certifies that their contribution to this work meets the standards of the International Committee of Medical Journal Editors.

## Funding

This project is funded by the National Institute for Health and Care Research, Public Health Research programme (grant no. 129885). The views expressed here are those of the author(s) and not necessarily those of the National Institute for Health and Care Researchor the Department of Health and Social Care.

## Conflicts of Interest

The authors declare no conflicts of interest.

## Data Availability

The data that support the findings of this study are available from the corresponding author upon reasonable request.

## Endnotes

^1^
Including both direct quotes and paraphrasing.
